# Draft Genome Sequences of *Trichophyton rubrum* CMCC(F)T_1i_ and *Trichophyton violaceum* CMCC(F)T_3l_ by Illumina 2000 and Pacific Biosciences

**DOI:** 10.1128/genomeA.00920-17

**Published:** 2017-09-28

**Authors:** Ping Zhan, Sybren de Hoog, Weida Liu

**Affiliations:** a Department of Mycology, Institute of Dermatology, Chinese Academy of Medical Science & Peking Union Medical College, Nanjing, China; b Dermatology Hospital of Jiangxi Province, Dermatology Institute of Jiangxi Province, Nanchang, China; c Westerdijk Fungal Biodiversity Institute, Utrecht, The Netherlands

## Abstract

One strain of *Trichophyton rubrum* CMCC(F)T_1i_ (=CBS 139224) isolated from onychomycosis and one strain of *Trichophyton violaceum* CMCC(F)T_3l_ (=CBS 141829) isolated from tinea capitis in China were whole-genome sequenced by Illumina/Solexa, while the former was also sequenced by Pacific Biosciences sequencing in parallel.

## GENOME ANNOUNCEMENT

*Trichophyton rubrum* and *Trichophyton violaceum* are the most common siblings in the *T. rubrum* complex (1). They are highly conserved at the sequencing level and have quite different phenotypes (1, 2). To explore the different pathogenicity of these two dermatophytes, we whole-genome sequenced one strain of *T. rubrum* CMCC(F)T_1i_ and one strain of *T. violaceum* CMCC(F)T_3l_ in South China by Illumina/Solexa, while the former was sequenced by PacBio RS in parallel.

The strains were cultured in solid Sabouraud glucose agar and incubated at 27°C for 7 to 20 days. Genomic DNA was extracted using the EZNA fungal DNA kit (Omega, USA).

For Illumina sequencing, 5-μg genomic DNA was used in the sequencing library construction. Paired-end libraries with insert sizes of ∼300 bp were constructed using the AIR paired-end DNA sequencing kit (Bioscientific). Subsequently, 100 bp at each end was sequenced using Illumina Hiseq2000. The *T. violaceum* genome was assembled using Velvet assembler (v1.2.09), and contigs with a length less than 200 bp were discarded to get reliable assembled results.

For Pacific Biosciences sequencing, 8- to 10-kb insert whole-genome shotgun libraries were generated and sequenced on a Pacific Biosciences RS instrument using standard methods. The complete genome sequence of strain *T. rubrum* was assembled using both the PacBio reads and Illumina reads. The assembly was produced first using a hybrid *de novo* assembly solution modified by Koren et al., in which a de Bruijn-based assembly algorithm and a CLR reads correction algorithm were integrated in the PacBioToCA pipeline in the Celera assembler (3, 4). The last circular step was checked and finished manually. The final assembly generated a circular genome sequence with no existing gap.

Identification of predicted coding sequences (CDSs) was performed using AUGUSTUS v3.0.1 (http://bioinf.uni-greifswald.de/augustus/). Open reading frames (ORFs) with less than 300 bp were discarded. Then, the remaining ORFs were queried against the nonredundant (NR) NCBI, SwissProt (http://uniprot.org), KEGG (http://www.genome.jp/kegg/), and COG (http://www.ncbi.nlm.nih.gov/COG) databases to do functional annotation.

The third-generation sequencing technique can supply long, perfect sequences, as well as accurate mitochondrial structure. *T. rubrum* obtained 19 superscaffolds (scaffold_50_, 2,198,313 bp) with a whole-genome length of 22,289,584 bp, while *T. violaceum* obtained 278 scaffolds (scaffold_50_, 1335,347 bp) with 23,310,379 bp as the whole length. The overall G+C content of the entire genome is 48.34%, and the *N* rates are as low as 0.055% for *T. rubrum* and 47.22% and 0.76% for *T. violaceum*, respectively. A total of 7,170 genes were predicted for *T. rubrum* and 7,415 for *T. violaceum*. The two genomes show a quite high genome similarity and synteny, with 99.38% identity at the nucleic acid level. As for genes, there were only 18 different CDSs altogether.

This is the first report of the whole-genome sequence of *T. violaceum* and the first application of PacBio RS for dermatophyte genome sequencing. These high-quality sequences and subsequent comparative genomic analysis might provide a better understanding of the pathogenicity and diversity of *T. rubrum* and *T. violaceum* at the genome level.

### Accession number(s).

This whole-genome shotgun project has been deposited in DDBJ/EMBL/GenBank under the accession numbers LHPM00000000 for *T. rubrum* CMCC(F)T_1i_ and LHPN00000000 for *T. violaceum* CMCC(F)T_3l_.
